# Applications of Large Language Models in Medical Research: From Systematic Reviews to Clinical Studies

**DOI:** 10.3390/bioengineering13030365

**Published:** 2026-03-20

**Authors:** Eun Jeong Gong, Chang Seok Bang, Yong Seok Shin

**Affiliations:** 1 Department of Internal Medicine, Hallym University College of Medicine, Chuncheon 24252, Republic of Korea; 2 Institute for Liver and Digestive Diseases, Hallym University, Chuncheon 24252, Republic of Korea; 3 Institute of New Frontier Research, Hallym University College of Medicine, Chuncheon 24252, Republic of Korea

**Keywords:** large language models, ChatGPT, GPT-4, systematic review, medical research, artificial intelligence, prompt engineering, evidence synthesis

## Abstract

**Background**: Large Language Models (LLMs) are reshaping medical research workflows. Objective: This narrative review synthesizes evidence on LLM applications across systematic reviews, scientific writing, and clinical research. **Methods**: We reviewed literature from 2023–2025 examining LLM applications in medical research, identified through PubMed, Scopus, Web of Science, arXiv, medRxiv, and Google Scholar. Studies reporting empirical findings, methodological evaluations, or systematic analyses of LLM applications were included; editorials and commentaries without empirical data were excluded. **Results**: In systematic reviews, LLMs achieve 80–94% data extraction accuracy and 40% reduction in screening workload, but show only slight-to-moderate agreement (κ = 0.16–0.43) in risk-of-bias assessment. In scientific writing, hallucination rates of 47–55% for fabricated references and over 90% prevalence of demographic bias require rigorous verification. For clinical research, LLMs assist with statistical coding and protocol development but require human validation. Critically, excessive reliance on automated tools may cause cognitive offloading that compromises analytical capabilities. **Conclusions**: LLMs are powerful but unstable tools requiring constant verification. Success depends on maintaining human-in-the-loop approaches that preserve critical thinking while leveraging AI efficiency.

## 1. Introduction

Since ChatGPT’s public release in November 2022, large language models (LLMs)—transformer-based neural networks capable of understanding context, following complex instructions, and generating human-like text—have generated substantial interest within the medical research community [1,2,3,4,5]. These models, trained on massive datasets and containing billions of parameters, offer potential solutions to longstanding challenges in medical research, particularly the labor-intensive nature of evidence synthesis and the exponential growth of medical literature [6,7].

The systematic review (SR) process presents significant challenges. Reviews require an average of 67.3 weeks from protocol registration to publication, with research teams investing hundreds of person-hours in screening thousands of abstracts, extracting data, and synthesizing findings [8]. With PubMed adding over 1.5 million citations annually, the volume of literature has exceeded human capacity for full synthesis using traditional methods [9]. Given these challenges, LLMs may help researchers manage the growing volume of literature, though human expertise remains essential for quality control [10].

Recent surveys of mental health researchers found that 69.5% have used LLMs, though fewer than 15% employ them for complex analytical tasks such as data analysis or study design [11]. This gap between interest and implementation stems from multiple factors, including uncertainty about best practices, concerns about accuracy, a lack of institutional guidance, and limited practical implementation frameworks [12]. Early implementations have shown mixed results, highlighting the need for evidence-based guidance on appropriate use cases, validation methods, and ethical considerations.

The phenomenon of hallucination, where LLMs generate plausible but entirely fabricated information, poses significant risks in medical contexts where accuracy directly affects patient care decisions [5,13]. Additionally, questions about transparency, reproducibility, and accountability challenge traditional notions of authorship and scientific responsibility [14]. These concerns have prompted major medical journals and professional organizations to develop guidelines for artificial intelligence (AI) use in research, though standards remain heterogeneous and evolving [15].

This narrative review assesses current evidence on LLM applications across three domains: SR methodology, narrative review composition, and clinical research applications, identifying strengths, limitations, and optimal integration strategies.

Although numerous reviews have addressed LLM applications in medicine between 2023 and 2025, existing publications tend to focus on individual domains: clinical applications [2,16], SR automation [17,18], scientific writing [19], or clinical trials [20]. No existing review integrates all three domains—SR methodology, scientific writing, and clinical research applications—under a unified, researcher-centric framework spanning the entire research lifecycle. Our review addresses this gap by providing an integrative synthesis that connects these domains and introduces several novel conceptual contributions, including the “cognitive offloading paradox” with supporting neuroscience evidence, the concepts of “never-skilling” and “mis-skilling” in research training, and the “paywall blind spot” as a systematic limitation of LLM training data for evidence synthesis.

We chose a narrative rather than a systematic review format for several methodological reasons. The LLM field evolves on a timescale of weeks, making systematic review protocols impractical for capturing current developments [21]. The literature we synthesize spans computational experiments, clinical pilots, theoretical analyses, and conference proceedings—sources that cannot be meaningfully combined under a single PICO framework. Furthermore, our integrative purpose—connecting disparate domains under a unified framework—requires the interpretive flexibility that narrative synthesis affords [22,23]. The SANRA (Scale for the Assessment of Narrative Review Articles) framework [24] provides the appropriate quality assessment standard for this format.

### 1.1. Search Strategy and Study Selection

This narrative review was conducted by searching PubMed, Scopus, Web of Science, arXiv, medRxiv, and Google Scholar for articles published between January 2023 and May 2025. The primary search terms included “large language model,” “ChatGPT,” “GPT-4,” “LLM,” and “generative AI,” combined with domain-specific terms including “systematic review,” “meta-analysis,” “medical research,” “clinical trial,” “scientific writing,” “data extraction,” and “evidence synthesis.” Studies were included if they reported original empirical findings, methodological evaluations, or systematic analyses of LLM applications in medical research contexts. Editorials, commentaries, and opinion pieces without empirical data were excluded unless they provided novel conceptual frameworks. Reference lists of included studies and relevant review articles were manually screened to identify additional eligible publications. As a narrative review, we aimed for comprehensive but not exhaustive coverage, prioritizing studies with empirical performance data and those reporting validation metrics.

### 1.2. Large Language Models in Systematic Reviews

#### Literature Search Strategy Generation

The development of thorough search strategies represents the foundational step in SR methodology, requiring expertise in controlled vocabularies, Boolean operators, and database-specific syntax. Recent investigations have examined whether LLMs can assist in generating these complex queries, with mixed results that highlight both potential utility and significant limitations.

Wang et al. conducted an early systematic evaluation of ChatGPT’s ability to generate Boolean queries for SR literature searches [25]. Their findings revealed a trade-off: LLM-generated queries demonstrated high precision but marked reduced recall compared to expert-crafted strategies. This precision-recall imbalance poses particular concerns for SRs, where comprehensive retrieval is paramount to minimizing selection bias. The application of guided chain-of-thought (CoT) prompting improved F1 scores dramatically from 0.077 to 0.517, suggesting that sophisticated prompt engineering can partially mitigate these limitations [25].

Subsequent research has further characterized the strengths and weaknesses of LLM-generated search strategies. Yu and colleagues, applying PRESS (Peer Review of Electronic Search Strategies) guidelines for evaluation, found that GPT-4 significantly outperformed GPT-3.5 in search strategy generation, particularly in the appropriate inclusion of Medical Subject Headings (MeSH) terms [26]. However, systematic deficiencies persist across models. An evidence summary by Parisi and Sutton identified that LLM-generated strategies frequently fail to incorporate synonymous entry terms, miss clinical practice jargon, incorrectly group acronyms, insert unjustified date limitations, and, critically, omit validated study design filters for identifying randomized controlled trials (RCTs) [27].

A critical limitation is that LLMs cannot directly execute searches across bibliographic databases such as PubMed, Embase, or the Cochrane Library. They can only generate query strings that human researchers should then adapt and run in each database’s specific interface. Furthermore, LLM-generated strategies rarely employ advanced search techniques such as truncation, wildcards, or proximity operators that information specialists routinely use to maximize retrieval [27]. Given these constraints, current evidence suggests that LLMs may serve as useful starting points for search strategy development but cannot replace the expertise of trained medical librarians or information specialists. Human validation remains essential before implementing any LLM-generated search strategy in an SR protocol. Importantly, the lack of standardized benchmarks for evaluating LLM-generated search strategies against expert-crafted ones limits the generalizability of current findings, and most existing evaluations assess only a single LLM version, making it difficult to track performance trajectories across rapidly evolving model iterations.

### 1.3. Literature Screening and Study Selection

The application of LLMs to literature screening represents one of the most mature use cases in SR methodology. Traditional screening, which requires assessing thousands of abstracts against predetermined inclusion criteria, consumes approximately 30–40% of total review time while being prone to human error, fatigue, and inconsistency [28]. The promise of LLM assistance lies not just in time savings but also in the potential to apply criteria more consistently and to process volumes of literature that would overwhelm human reviewers [29].

Studies examining LLM performance in the literature screening showed mixed results. Issaiy et al. tested ChatGPT on 1198 radiology abstracts from three subfields and reported 95% sensitivity [30]. The model correctly excluded over 50% of irrelevant citations without missing any eligible studies. Structured prompts outperformed narrative instructions. This suggests LLMs handle step-by-step decision-making more effectively than overall assessments [30]. The development of more advanced approaches has yielded improved results. The LARS-GPT system developed by Cai et al. uses a more sophisticated dual-phase screening approach [31]. It first identifies studies with high confidence as clearly irrelevant, then flags borderline cases for human review. This strategy achieved recall rates above 0.9 while reducing human screening workload by 40% [31].

Significant limitations have also been identified. Khraisha Q and colleagues conducted perhaps the most thorough evaluation to date, testing GPT-4 across multiple languages and publication types [32]. Their findings revealed dramatic performance degradation for non-English texts, with data extraction sensitivity dropping from 75% for English articles to just 36% for non-English publications [32]. This language bias represents a critical limitation for comprehensive SRs that aim to minimize it, particularly given that important research from non-English-speaking countries may be systematically excluded if reviewers rely too heavily on LLM assistance.

Prompt engineering significantly affects screening performance, though effects remain unpredictable. Kohandel Gargari et al. found inherent trade-offs between sensitivity and specificity, with some presumably beneficial modifications (e.g., expert role assignment) paradoxically degrading accuracy [33]. Prompt selection should, therefore, align with review priorities—sensitivity for comprehensive reviews, specificity when workload reduction is paramount [33].

Model parameters such as temperature settings influence output consistency, though their impact on screening accuracy remains underexplored. Most screening studies use temperature = 0 for reproducibility, as this eliminates response variation between identical queries [34]. However, recent evidence suggests that temperatures between 0.0 and 1.0 produce statistically equivalent classification accuracy, with significant degradation occurring only above 1.5 [35]. The practical recommendation is to set temperature = 0 and document this choice explicitly for methodological transparency.

Table 1 summarizes recent evidence on LLM performance in SR screening. As these studies demonstrate, performance varies considerably across models, with distinct trade-offs between sensitivity and specificity [36]. When combining outputs using an ensemble method that included citations flagged by any model, sensitivity improved while specificity decreased, suggesting that strategically integrating multiple LLMs can enhance screening coverage at the expense of increased false positives requiring human review [36]. Similarly, multi-agent collaborative frameworks—where distinct LLMs provide initial analyses, review each other’s outputs, and converge on consensus decisions through majority voting—have shown promising results in diagnostic accuracy tasks, with one study reporting 98% accuracy among the top three diagnoses compared to 71–96% for individual models [37]. The concept of an “LLM Council,” popularized by Karpathy, extends this approach by having multiple models anonymously evaluate and rank each other’s responses before a designated “chairman” model synthesizes the final answer, though this method may favor verbosity over conciseness [38]. These complementary strengths of different models suggest significant potential for multi-model approaches in high-stakes SRs where missing relevant studies carry significant consequences [39].

### 1.4. Data Extraction and Evidence Synthesis

The transition from screening to data extraction represents a major increase in complexity, requiring LLMs not only to identify relevant information within lengthy and often poorly structured texts but also to accurately transcribe specific values, understand statistical presentations, and maintain consistency across heterogeneous reporting styles. The challenge is compounded by the variety of data types encountered in medical research, from simple demographic information to complex statistical analyses and subtle clinical outcomes [40].

Recent evaluations of LLM performance in data extraction reveal both impressive capabilities and concerning limitations (Table 2) [41,42]. Single LLM approaches achieve approximately 80% overall accuracy, with variation across domains (82% in clinical, 72% in social science studies) [41]. Simple data elements, such as participant characteristics, interventions, and study locations, are typically extracted with high accuracy (80–90%). Meanwhile, more complex information, such as outcomes, causal inference methods, and study design, shows notably lower performance [41]. A collaborative dual-LLM approach using GPT-4-turbo and Claude-3-Opus significantly improves accuracy to 94% when both models agree, while reducing hallucination rates from approximately 2.5% to 0.25% [42]. However, performance drops markedly for non-English texts, with sensitivity falling to 36% [32].

Document format significantly affects extraction accuracy. Portable document format (PDF) parsing quality was identified as the primary determinant of success, with GPT-4 achieving only 68.8% accuracy using automated PDF parsing versus 100% with manually selected text [43,44]. While multimodal vision-language models can process documents without separate optical character recognition (OCR) steps, current models still exhibit notable limitations requiring careful validation [45,46,47].

Language barriers present another significant challenge, with GPT-4’s data extraction sensitivity dropping from 75% for English articles to 36% for non-English publications [32]. This has important implications for SRs aiming for coverage of global literature. Teams must either restrict their reviews to English-language publications—potentially introducing language bias—or maintain significant human resources for processing non- English literature.

Several research groups have developed structured extraction protocols to improve performance. Khan et al. implemented a collaborative two-LLM approach using GPT-4-turbo and Claude-3-Opus in parallel, with consensus between models serving as a quality filter that substantially outperformed single-model extraction [42]. Pre-specified extraction schemas with standardized prompts have shown 95–97% test–retest reliability across multiple extraction rounds [44]. Task chunking—separating complex extractions into smaller independent tasks—has been specifically recommended for improving numerical data accuracy [48].

The phenomenon of hallucinated data in extraction tasks represents a critical concern. Motzfeldt Jensen and colleagues identified a 5.2% false-data rate where LLMs fabricated outcome values rather than reporting them as missing [49]. When information was not explicitly reported in papers, reproducibility decreased from 94.1% to 77.2% as models attempted to infer data based on training patterns. Such systematic errors would directly corrupt meta-analytic effect estimates, emphasizing the importance of human verification, particularly for outcome data and statistical results, rather than complete automation of the extraction process. These limitations are particularly concerning, given that most evaluation studies have been conducted in controlled settings and published in well-structured papers; real-world extraction from diverse source types (conference abstracts, government reports, gray literature) is likely to yield substantially lower accuracy than reported benchmarks suggest.

### 1.5. Risk-of-Bias Assessment

Risk-of-bias assessment is one of the most challenging applications of LLMs in SRs, requiring not only information extraction but also careful judgment about study conduct and reporting quality. The subjective nature of many bias assessments, combined with the need to infer information that may not be explicitly stated, creates unique challenges that test the limits of current LLM capabilities. Recent studies evaluating LLM performance on standardized risk-of-bias tools have revealed notable limitations, with agreement between AI and human reviewers remaining modest across multiple evaluation frameworks [12].

Recent studies have evaluated LLM performance in risk-of-bias assessment using the Cochrane RoB 2 tool for RCTs. Pitre and colleagues assessed ChatGPT-4 against Cochrane author judgments across 157 RCTs from 34 reviews, finding only slight agreement with Cohen’s κ of 0.16 for overall risk-of-bias assessment [50]. Similarly, Kuitunen et al. evaluated ChatGPT-4o on 100 RCTs from high-impact journals and reported slight agreement for the overall assessment (κ = 0.24) and the randomization domain (κ = 0.31), with no agreement to poor agreement in the other domains [51]. A subsequent study by the same group analyzing 61 neonatal RCTs reported moderate overall agreement (κ = 0.43) [52]. The available evidence suggests variable performance across different bias domains, with LLMs showing better performance on objective criteria, such as allocation concealment (κ = 0.73), compared to subjective judgments, such as incomplete outcome data assessment (κ = −0.03) [52].

These performance gaps likely reflect fundamental limitations in how current LLMs process implicit methodological information—risk-of-bias judgments frequently require inferring study conduct from what is not reported, a task that challenges models trained primarily on explicit textual patterns [53]. Nevertheless, several promising strategies for improvement have emerged. Structured prompting strategies incorporating domain-specific guidelines have demonstrated substantially higher accuracy, with one study achieving 89.5% correct assessment rates using well-designed prompts compared to lower performance with basic approaches [54]. Human-in-the-loop workflows that combine LLM screening with expert verification appear particularly promising, reducing assessment time by over 90% while maintaining accuracy comparable to conventional methods [55]. Future advances may require domain-specific fine-tuning on large corpora of completed Cochrane assessments, development of retrieval-augmented approaches that can access methodological guidance documents, and better handling of the contextual reasoning required for subjective domains. Until such advances materialize, LLMs are best positioned as assistive tools that accelerate the mechanical aspects of risk-of-bias assessment while preserving human judgment for the complex interpretations that define rigorous evidence synthesis.

### 1.6. Large Language Models in Narrative Review Writing

#### Augmenting Scientific Writing

The application of LLMs to scientific writing extends far beyond simple grammar correction, encompassing advanced capabilities in structure development, argumentation refinement, and stylistic consistency. For narrative reviews, which require synthesizing diverse literature into coherent narratives while maintaining author voice and critical perspective, LLMs offer both promising opportunities and unique challenges [56].

Non-native speakers particularly benefit from LLM assistance [57]. With over 80% of indexed scientific journals publishing in English, non-native speakers face significant disadvantages in manuscript preparation, peer review communication, and career advancement [57]. LLMs can help bridge this gap by improving grammatical accuracy, sentence structure, and overall clarity, potentially democratizing access to international publication venues [5,58,59].

The process of scientific writing involves multiple layers of complexity that LLMs address through different mechanisms. At the most basic level, these models excel at correcting grammatical errors and improving sentence structure, tasks that require understanding of both linguistic rules and scientific conventions [58]. Their capabilities extend to higher-order concerns such as logical flow and argumentative coherence, though their effectiveness in these areas depends heavily on the quality of human guidance and the specific requirements of the writing task [60].

Table 3 summarizes the common issues identified in LLM-generated scientific text along with detection methods and mitigation strategies. Hallucination poses serious risks in scientific writing. Analysis of LLM-generated medical texts reveals high rates of fabricated references. Bhattacharyya et al. found that among 115 references generated by ChatGPT-3.5, 47% were completely fabricated, 46% were authentic but contained inaccuracies, and only 7% were both authentic and accurate [48]. Walters and Brainard reported even more variation across model versions: GPT-3.5 produced 55% fabricated citations compared to 18% for GPT-4, demonstrating that newer models show improvement but remain unreliable [61]. These fabricated references often appear entirely plausible, making detection challenging without systematic verification [62]. Additionally, LLMs may misrepresent study findings when paraphrasing, introduce inaccurate statistical claims, or generate content that sounds authoritative but lacks a factual basis [63].

The development of verification protocols has become essential for the safe implementation of LLM-assisted writing. These protocols typically involve multiple layers of checking, beginning with automated verification of references against bibliographic databases, followed by validation of numerical claims against original sources, and culminating in expert review of technical accuracy [63]. While time-consuming, such verification is necessary to maintain scientific integrity and prevent the propagation of errors through the literature. A critical concern is that the verification burden may paradoxically negate the time savings offered by LLM-assisted writing, particularly for non-expert users who may lack the domain knowledge to identify subtle inaccuracies or misrepresentations of study findings. The net efficiency gain, therefore, depends heavily on the user’s baseline expertise, a factor that remains underexplored in the existing literature.

The challenge of maintaining technical accuracy while improving readability represents a persistent tension in LLM-assisted writing. Models may oversimplify complex concepts in pursuit of clarity, potentially altering meaning or omitting important nuances [64]. This tendency requires careful human oversight to ensure that improvements in readability do not compromise scientific precision. Researchers must remain vigilant for subtle changes in meaning that could affect the interpretation of findings or the validity of conclusions.

Iterative, multi-stage prompting outperforms single-prompt generation for writing tasks. The Self-Refine approach—where LLMs generate output, provide self-feedback, then iteratively refine—improved quality by approximately 20% compared to one-step generation [66]. Similarly, prompt chaining (sequential drafting, critiquing, and refining) outperformed single-stepwise prompts in 77 of 100 text summarization evaluations, as stepwise prompts often produced “simulated refinement”, where models intentionally introduced errors to subsequently correct them [67]. These findings support decomposing writing tasks into sequential stages to allow human verification at each step.

### 1.7. Literature Synthesis and Thematic Analysis

The synthesis of disparate research findings into coherent narratives represents one of the most intellectually demanding aspects of review writing. LLMs show strong capabilities in identifying patterns across large bodies of literature, though their effectiveness depends critically on how they are deployed and supervised [65]. The ability to process and synthesize information from multiple sources simultaneously enables the identification of connections that might not be apparent when reading studies sequentially.

Studies comparing LLM-generated analyses with those produced by experienced researchers show considerable overlap in identified themes, though with important differences in depth and point [68]. LLMs tend to identify surface-level patterns effectively but may miss subtle theoretical connections or methodological implications that experienced researchers would recognize. They also tend to impose coherence where none exists, potentially obscuring genuine controversies or contradictions in the literature.

The iterative refinement of narratives through human-AI collaboration appears more effective than either approach alone. This collaborative model uses the LLM’s ability to process large volumes of information while maintaining the human researcher’s critical judgment and domain expertise [60]. For multi-author projects, LLMs may assist with harmonizing writing styles, standardizing terminology, and maintaining consistency across sections, though empirical validation of these applications remains limited.

### 1.8. Large Language Models in Clinical Research and Data Analysis

#### Statistical Programming and Analysis

The application of LLMs to statistical programming represents a particularly promising yet complex domain. Recent evaluations demonstrate that LLM-generated statistical code achieves 32–93% accuracy depending on prompt specificity and task complexity [69]. For descriptive statistics, LLMs achieve near-perfect accuracy, but performance declines substantially for complex analyses requiring assumption verification and appropriate method selection [69,70].

Studies comparing LLM-generated analyses with traditional software (SAS, SPSS, R) reveal consistent results for basic calculations but significant discrepancies for advanced methods [70]. ChatGPT-4 cannot autonomously select appropriate analyses without specific user instructions, and complex procedures such as Cox regression and MANOVA show notable error rates, including miscalculated degrees of freedom and implausible confidence intervals [71,72]. In survival analysis, LLMs consistently underestimate required sample sizes due to systematic errors in applying statistical formulas. Meanwhile, meta-analyses show high variability and inappropriate model selection based solely on heterogeneity thresholds [71].

The development of statistical analysis plans (SAPs) through LLM assistance shows preliminary promise, with case studies demonstrating acceptable SAP generation within 15 min [73]. However, rigorous validation comparing LLM-generated SAPs with those from human biostatisticians remains lacking. A particular risk is that researchers without strong statistical backgrounds may accept LLM-generated analyses uncritically, potentially propagating errors in assumption checking, model selection, and result interpretation that could compromise study conclusions. Table 4 summarizes key considerations for LLM-assisted statistical programming based on empirical evidence [69,71,74,75,76,77].

### 1.9. Clinical Data Processing

The extraction and processing of clinical data from electronic health records (EHRs) represents an area where LLMs have demonstrated strong capabilities. Recent studies showed that adapted LLMs can outperform medical experts in clinical text summarization, with GPT-4 summaries rated equivalent or superior to expert summaries in 81% of evaluations [78]. LLMs showed promise for various EHR-related tasks, including diagnosis extraction, medication reconciliation, and outcome ascertainment, with GPT-4 producing the highest-quality phenotyping algorithms when generating executable Structured Query Language (SQL) queries for patient identification [79].

The challenge of maintaining privacy while using LLM capabilities has led to the development of specialized approaches for handling sensitive data. These typically involve complete de-identification before processing, use of locally deployed models that never transmit data externally, and careful audit logging of all data access [80]. Privacy-preserving frameworks using open-source models such as Llama 2 have achieved 100% sensitivity and 96% specificity for clinical information extraction while running entirely on-premises, eliminating the need for cloud data transfer and addressing GDPR/HIPAA compliance concerns [81].

Real-world clinical documentation presents unique challenges that test the limits of LLM capabilities. Clinical text often contains abbreviations, misspellings, negations, and implicit information that models may misinterpret. Recent work demonstrates GPT-4 can achieve 98% accuracy for clinical acronym disambiguation in zero-shot settings, though performance drops for non-English texts, and smaller models frequently produce hallucinations [82].

The temporal complexity of clinical data—with events across multiple encounters—poses additional challenges, though time-aware approaches have shown modest improvements in longitudinal reasoning [83]. For cohort construction and clinical trial matching, LLMs show potential for initial screening, though current systems tend toward overly restrictive or overly broad phenotyping [84].

### 1.10. Clinical Trial Protocol Development

LLMs are reshaping clinical trial protocol development across multiple dimensions. In a protocol-writing study, Markey et al. evaluated GPT-4’s ability to generate protocol sections, including endpoints and eligibility criteria. Off-the-shelf GPT-4 performed well on content relevance and medical terminology (scores > 80%) but showed limited performance in clinical thinking/logic and transparency/references (scores 40% or less). However, retrieval-augmented generation (RAG) incorporating regulatory guidance and ClinicalTrials.gov data markedly improved these weaker dimensions to approximately 80%, demonstrating that hybrid architectures greatly enhance practical usability for protocol writing [85].

For patient-facing materials, ensuring accessibility remains critical. Ali et al. demonstrated that GPT-4 could reduce the reading level of consent forms from the college freshman level to the eighth-grade level while maintaining completeness and legal validity. Their AI-human collaborative framework—validated by physicians and medical malpractice attorneys—achieved sixth-grade readability for procedure-specific forms while achieving perfect scores on consent quality metrics [86]. Though focused on surgical consents, this approach extends to clinical trial informed consent documents facing similar literacy barriers.

SAP generation and adaptive trial design represent emerging applications, though rigorous validation studies remain limited. The complexity of regulatory requirements and the need for methodological precision demand careful human oversight in these domains.

Patient-trial matching presents perhaps the most mature LLM application. Jin et al. developed TrialGPT, an end-to-end framework that retrieves candidate trials, predicts criterion-level eligibility with explanations, and ranks trials for patients. TrialGPT achieved 87.3% accuracy on eligibility predictions and reduced clinician screening time by 42.6% in user studies, while recalling over 90% of relevant trials from just 6% of initial collections [84]. This automated matching addresses a critical bottleneck in trial recruitment.

## 2. Methodological Considerations

### 2.1. Prompt Engineering and Optimization

The effectiveness of LLMs in medical research depends critically on prompt engineering, yet optimal strategies remain largely empirical. A scoping review of 114 studies identified prompt design as the most prevalent approach, though terminology remains inconsistent across the field [87]. Structured prompts with explicit role definition, clear output specifications, and step-by-step instructions generally improve performance, but “prompt brittleness” means even minor wording changes can markedly alter outputs [74,88].

CoT prompting shows particular promise for clinical reasoning, with o1-mini achieving 88.4% accuracy on clinical question-answering tasks [89]. However, CoT benefits primarily large models (≈100 B parameters); smaller models often produce illogical reasoning that degrades performance [90]. Self-consistency sampling improved MedQA accuracy by over 7% but decreased performance on other datasets, highlighting the need for task-specific validation [91].

Temperature settings have a smaller impact than commonly assumed. GPT-4o maintained consistent accuracy (98.7–99.0%) across temperatures 0.0–1.5, with degradation only above 1.75 [35]. For reproducibility, temperature = 0 is recommended, though true determinism remains elusive due to hardware-level variations.

### 2.2. Validation and Quality Assurance

Robust validation is essential for LLM-assisted research. The MI-CLEAR-LLM checklist identifies six critical reporting items affecting reproducibility: LLM identification, stochasticity handling, prompt documentation, prompt structuring, optimization details, and test data independence [92]. However, an analysis of studies published in top medical journals found that only 15.1% adequately reported stochasticity handling [75]. Common error types include reference hallucination, numerical transposition, and context misunderstanding [93,94]. Multi-layered validation, combining automated checks and expert review, can catch most errors before they propagate. Reproducibility remains challenging: model versions change, outputs vary stochastically, and prompt sensitivity means minor variations produce different results [95].

### 2.3. Ethical and Regulatory Considerations

#### Publication Ethics and Attribution

Major medical journals and organizations have established policies requiring disclosure of AI use, though requirements vary considerably [15,96]. The consensus that LLMs cannot be listed as authors reflects fundamental principles about responsibility and accountability—AI tools cannot fulfill ICMJE authorship criteria requiring intellectual contribution and responsibility for accuracy [96]. As of 2024, over 80% of publisher policies require disclosure statements when AI is used in manuscript preparation [97].

Questions remain about appropriate attribution when LLMs contribute substantially to analysis or writing. The challenge of maintaining transparency while protecting intellectual property creates tension: some journals require submission of complete prompts and interaction logs, potentially revealing proprietary methods or sensitive information [98]. Balancing transparency with practical considerations remains an ongoing challenge for journals and researchers.

### 2.4. Data Privacy and Security

The use of LLMs with clinical data raises critical privacy concerns requiring careful management [99]. Regulatory frameworks such as HIPAA/GDPR impose strict requirements that may be incompatible with commercial LLM services, while local deployment requires technical expertise not widely available [100,101]. International variations in privacy regulations add complexity, and developing compliant workflows that satisfy multiple regulatory frameworks remains challenging [102,103].

### 2.5. Access Limitations and Information Bias

LLMs are predominantly trained on web-crawled data, with academic content comprising only 2–5% of training tokens. The Pile dataset, used by multiple open-source models, explicitly includes only open-access sources such as PubMed Central and arXiv, with no paywalled journal content [104]. Common Crawl, which provides over 80% of GPT-3’s training tokens, systematically underrepresents academic literature through authentication barriers that prevent access to subscription content [105]. This creates a “paywall blind spot” where LLMs may have limited exposure to methodological details in premium publications from Elsevier, Springer Nature, and Wiley. Researchers must supplement LLM-assisted synthesis with subscription database queries to ensure comprehensive literature coverage.

### 2.6. Bias and Fairness

Systematic biases in LLM outputs pose risks for medical research that could perpetuate health disparities. A SR found that over 90% of studies identified demographic biases in medical LLMs [106]. These biases manifest in clinically consequential ways: ChatGPT, GPT-4, and Claude propagate debunked race-based medicine, including false claims about racial differences in kidney function and pain thresholds [107]. GPT-4 was more likely to rate Black patients as abusing opioids when presented with identical clinical information [108]. Mitigation strategies—including bias education prompts and diverse prompt testing—show promise but remain insufficiently validated [109]. Researchers should implement systematic demographic auditing before deploying LLMs in clinical applications.

### 2.7. Integrating Scientific Integrity into LLM Workflows

Minimizing risks associated with bias and model inaccuracy requires embedding principles of scientific integrity at every stage of LLM-assisted research workflows. We propose a five-component framework. First, transparency and reproducibility should be ensured through mandatory documentation of model version, temperature settings, complete prompts, and API parameters, as recommended by the MI-CLEAR-LLM checklist [92]; notably, only 15.1% of studies in top medical journals adequately reported stochasticity handling [75]. Second, multi-layered verification should combine automated checking (reference verification against bibliographic databases), cross-model consensus (dual-LLM approaches reducing hallucination from approximately 2.5% to 0.25% [42]), and expert review as the final arbiter. Third, systematic bias auditing should be implemented as a standard protocol before any LLM deployment in research, given that over 90% of studies have identified demographic biases in medical LLMs [106]. Fourth, institutions should establish human-in-the-loop governance that specifies which research tasks are appropriate for LLM assistance versus those that require fully human execution, addressing the dual risks of automation bias and automation neglect [110,111,112,113]. Fifth, research training programs should ensure foundational skills in manual literature screening, data extraction, and critical appraisal before introducing AI assistance, thereby preventing the “never-skilling” phenomenon in which trainees fail to develop independent analytical capabilities [114].

### 2.8. Limitations and the Human-AI Partnership

Despite impressive capabilities in pattern recognition and text generation, current LLMs exhibit limitations in logical reasoning and causal inference, which are critical for medical research. While excelling at identifying correlations across vast datasets, they struggle with counterfactual reasoning and may fail to recognize confounding factors—for instance, correctly identifying drug-outcome associations in observational data while missing why an RCT might yield different results [115]. This reasoning gap necessitates a “human in the loop” approach where researchers provide causal understanding while LLMs handle information synthesis.

Beyond limitations in reasoning, human-AI interaction challenges raise additional concerns. While automation bias—over-reliance on AI outputs—has received attention, the converse phenomenon of automation neglect poses equal risks in research contexts. Automation neglect occurs when experienced researchers dismiss AI recommendations due to overconfidence in their own judgment or distrust of AI systems [110,111,112]. In clinical AI studies, experts were significantly more likely than non-experts to ignore accurate AI recommendations, with up to 16% of correct outputs being dismissed [113]. In medical research, this may manifest as senior investigators rejecting valid LLM-identified studies during screening or dismissing accurate data extractions, potentially introducing systematic errors that paradoxically favor human fallibility over AI accuracy.

Excessive reliance on LLM-generated syntheses poses a subtle risk through cognitive offloading—delegating mental processes to external systems. Stadler et al. demonstrated this paradox experimentally: students using LLMs for scientific inquiry reported significantly lower cognitive load but produced arguments with significantly lower validity compared to those using traditional methods [116]. When researchers rely on automated “deep research” functions that provide pre-digested results, they may bypass the critical cognitive processes essential for developing expertise and recognizing novel patterns. Recent neuroscience research provides biological evidence for these concerns. A study tracking brain activity during AI-assisted cognitive tasks demonstrated that users showed up to 55% reduced neural connectivity in frequencies associated with deep thinking, with an impaired ability to recall content they had just produced—a phenomenon termed “cognitive debt”, where immediate convenience creates long-term cognitive costs [117]. Medical researchers should therefore maintain proficiency in traditional methods and actively engage with primary sources, recognizing that apparent efficiency gains may represent a trade-off against long-term analytical capability [118].

These concerns extend particularly to research training. The phenomenon of “never-skilling”—where trainees who learn SR methods exclusively with AI assistance fail to develop independent analytical capabilities—poses risks for the next generation of researchers [114]. Additionally, “mis-skilling,” where AI errors or biases are learned and perpetuated by trainees as correct methodology, may systematically compromise research quality. Just as clinical educators now advocate for periodic AI-free practice to preserve diagnostic competence, research training programs should ensure foundational skills in manual literature screening, data extraction, and critical appraisal before introducing AI assistance.

## 3. Research Question and Hypothesis Generation

LLMs show emerging potential for generating novel research questions and scientific hypotheses. By synthesizing patterns across vast bodies of literature, LLMs can identify knowledge gaps and propose testable hypotheses that might not be apparent to individual researchers. A recent study experimentally validated this capability: GPT-4 was tasked with hypothesizing novel synergistic drug combinations for breast cancer treatment, and laboratory experiments confirmed that 3 of 12 AI-generated hypotheses (25%) demonstrated synergy scores exceeding those of positive controls [119]. In a subsequent iterative round, 3 of 4 additional AI-suggested combinations also showed positive synergy. While these results suggest LLMs can serve as valuable sources of scientific hypotheses, concerns remain about their tendency to reinforce existing paradigms rather than proposing truly innovative directions, and validation comparing AI-generated research questions with expert-derived hypotheses remains limited.

## 4. Future Directions

Several technological advances promise to address current limitations. Multimodal models processing text, images, and structured data simultaneously will enable more comprehensive analysis of complex medical information [120]. Retrieval-augmented generation, combining LLM reasoning with real-time database access, could address concerns about hallucination and outdated information [121]. Specialized medical models trained on biomedical literature show promise for improved domain-specific performance, though validation frameworks and bias assessment remain essential [122].

The recent success of AI-discovered therapeutics, including the first AI-identified drug showing efficacy in Phase IIa trials, demonstrates that LLMs are transitioning from assistive tools to active partners in hypothesis generation [123]. Future applications may include autonomous experimental design, real-time adaptive trial modifications, and continuous evidence synthesis that automatically incorporates new findings. However, realizing this potential requires the development of explainable AI for medical research, the integration of causal reasoning capabilities, and ethical frameworks for attribution when AI contributes substantively to discovery [39,76,77].

### Emerging Open-Source and Cost-Effective Models

While this review has focused predominantly on GPT-series models—reflecting the composition of the published evidence base through May 2025—the rapid emergence of open-source alternatives with competitive performance at substantially lower cost represents a significant development for the democratization of AI-assisted medical research. DeepSeek-R1 (671 B parameters, mixture-of-experts architecture, MIT license) achieved 92% accuracy on USMLE questions, approaching GPT-4o’s 95% [124]. In 125 standardized patient cases, DeepSeek-R1 performed on par with GPT-4o in clinical decision-making tasks (*p* = 0.31) [125]. At approximately $0.28 per million input tokens—roughly 9–24 times cheaper than GPT-4o—and with open-weight deployment eliminating cloud data transfer requirements, DeepSeek addresses both cost and privacy barriers simultaneously.

Similarly, Qwen (Alibaba Cloud, Apache 2.0 license) demonstrated strong performance on Chinese-language medical tasks, achieving 88.9% accuracy on the Chinese National Nursing Licensing Examination compared to GPT-4o’s 80.7% [126]. However, on English-language medical benchmarks, Qwen generally trails GPT-4o (e.g., 0.57 vs. 0.73 accuracy in cancer genetic variant classification; [127]). Both models support local deployment, enabling institutions to process sensitive clinical data without cloud transmission—a critical advantage for HIPAA/GDPR compliance. However, important limitations remain: DeepSeek-R1 lacks native multimodal capability, generates verbose responses with increased latency, and its reasoning module does not consistently improve clinical performance over its base model. These emerging models underscore the need for review frameworks that transcend any single model’s capabilities and instead evaluate the general principles of human-AI collaboration in medical research.

## 5. Conclusions

This review synthesizes current evidence on LLM applications across SRs, scientific writing, and clinical research. LLMs demonstrate variable but promising performance: literature screening shows high sensitivity with substantial workload reduction, while tasks requiring subjective judgment, such as risk-of-bias assessment, remain insufficiently validated for standalone use. Hallucination and demographic bias represent critical concerns demanding rigorous verification protocols and systematic auditing before clinical deployment. The cognitive offloading paradox presents an underappreciated risk: while LLMs reduce cognitive burden and increase efficiency, excessive reliance may systematically weaken researchers’ analytical capabilities.

This review has several limitations. As a narrative review rather than a systematic review, our literature search and study selection, though structured, were not exhaustive. The rapid pace of LLM development means that some findings reviewed here may already be outdated. Publication bias toward positive results may overestimate LLM capabilities, and the heterogeneity of evaluation metrics across studies limits direct comparisons. Furthermore, most evidence derives from studies using proprietary commercial models (e.g., GPT-4), whose underlying architectures and training data are not fully transparent, limiting reproducibility and generalizability of findings.

**Figure 1:**
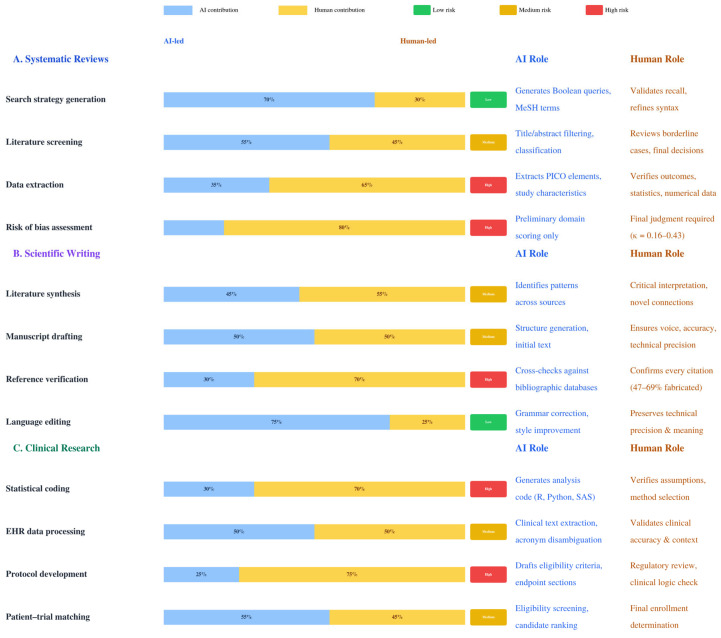
Recommended human–AI collaborative workflow framework for LLM applications across three medical research domains (systematic reviews, scientific writing, and clinical research). Each task is classified by risk level: low (LLM-led, acceptable with spot checks), medium (LLM-assisted, with systematic human review), or high (human judgment essential, with the LLM as a preliminary tool only). Core principles include universal verification, full documentation per MI-CLEAR-LLM guidelines [92], systematic bias auditing [106], and preservation of traditional methodological skills to prevent cognitive offloading [116,117].

We recommend a structured approach (Figure 1): start with low-risk applications, implement multi-layered validation, maintain reproducible settings, and preserve human judgment for tasks requiring causal reasoning. LLMs are powerful but inherently unstable instruments requiring constant calibration—success depends on researchers maintaining their roles as critical overseers rather than passive consumers of AI-generated content. In practice, this means adopting iterative, step-by-step refinement rather than expecting polished output from single prompts, and rigorously verifying every AI-generated citation and claim against primary sources.

## Figures and Tables

**Table 1:** Performance of LLMs in systematic review screening.

Study	Model(s)	Number of Studies	Sensitivity	Specificity	Key Findings
Oami et al. 2025 [36]	GPT-4o	16,669	0.85	0.97	Higher specificity, lower sensitivity
	Gemini 1.5 Pro		0.94	0.85	Higher sensitivity, lower specificity
	Claude 3.5 Sonnet		0.94	0.80	Higher sensitivity, lowest specificity
	Llama 3.3 70B		0.88	0.93	Trade-off: sensitivity vs. specificity
	Ensemble (OR rule)		Improved	Decreased	Trade-off: sensitivity vs. specificity
Matsui et al. 2024 [34]	GPT-4 (3-layer)	4527	0.81–0.88	0.86–1.00	Layered screening approach effective
	GPT-3.5 (3-layer)		0.69–0.75	0.95–0.98	Lower sensitivity than GPT-4
Guo et al. 2024 [39]	GPT-3.5/GPT-4	24,307	0.76	0.91	No pretraining required
Kohandel Gargari et al. 2024 [33]	GPT-3.5 Turbo	200	0.38–0.69	0.25–0.85	Prompt structure critical; trade-offs inevitable
Cai et al. 2025 [31]	LARS-GPT (multi-LLM)	N/A	>0.90	N/A	40% workload reduction with dual-phase approach
Khraisha et al. 2024 [32]	GPT-4	2421	0.75 (English)	N/A	Sensitivity drops to 0.36 for non-English texts

LLM, large language model; GPT, Generative Pre-trained Transformer; N/A, not applicable.

**Table 2:** LLM performance in data extraction for systematic reviews.

Extraction Approach	Performance	Key Findings
Overall extraction (single LLM) [41]	Accuracy ~80%	82% clinical, 80% animal, 72% social science studies
PICO elements [41]	Accuracy >80% (P, I, C), lower for O	Participants/Intervention well-extracted; Outcomes challenging
Collaborative dual-LLM (concordant) [42]	Accuracy 94%	GPT-4-turbo + Claude-3-Opus agreement; hallucination rate 0.25%
Single LLM (discordant cases) [42]	Accuracy 41–50%	GPT-4-turbo 41%, Claude-3-Opus 50%; hallucination rate ~2.5%
Non-English texts [32]	Sensitivity 36%	Significant performance drop in non-English literature
PDF-dependent extraction [43,44]	68.8–100%	Automated PDF parsing 68.8% vs. manual text selection 100%

LLM, large language model; GPT, Generative Pre-trained Transformer; P, participants; I, interventions; C, comparisons; O, outcomes.

**Table 3:** Common issues in LLM-generated scientific text.

Issue Type	Estimated Frequency	Detection Method	Mitigation Strategy
Fabricated references	18–55% (model-dependent) [48]	Database verification	Verify citations
Inaccurate citations	24–46% [48,61]	Original source check	Verify bibliographic details
Incorrect PMID	93% of papers [48]	PubMed verification	Cross-check all PMIDs
Oversimplification	Common (not quantified) [64]	Expert review	Maintain technical precision
Lost nuance	Common (not quantified) [65]	Domain expert check	Preserve complexity
Style homogenization	Common (not quantified)	AI detection tools, stylometric analysis	Maintain author voice, iterative refinement

LLM, large language model; PMID, PubMed Identifier.

**Table 4:** Key considerations for LLM-assisted statistical programming.

Consideration	Challenge	Evidence	Recommendation
Assumption checking	Often omitted without explicit prompting; 43.8% accuracy with basic prompts [69]	Fails normality verification, inappropriate test selection [76]	Always verify assumptions manually
Model selection	May choose inappropriate tests; incorrect method selection was the most common error (66%, n = 51 of 77 total errors) [69]	44% of errors involved knowledge recall (wrong test selection, statistical vs. causal method confusion) [77]	Require statistical expertise for selection
Complex designs	Poor performance on hierarchical models, survival analysis, or meta-analysis [71]	R code for survival analysis worked without corrections in 7/10 sessions [71]	Use only for simple analyses initially
Reproducibility	Identical prompts yield different results across sessions [71]	High variability in meta-analysis outputs [71]	Verify across multiple runs
Stochasticity reporting	Stochastic outputs even at temperature = 0; model version changes alter results [74]	Only 15.1% of studies adequately reported stochasticity handling [75]	Document per MI-CLEAR-LLM; use temperature = 0; archive model versions

LLM, large language model.

## Data Availability

The data are available upon request to the corresponding author. All investigators have access to the final dataset.
